# Demonstration of *Cutaneous Allodynia* in Association with Chronic Pelvic Pain

**DOI:** 10.3791/1232

**Published:** 2009-06-23

**Authors:** John Jarrell

**Affiliations:** Department of Obstetrics and Gynecology, Faculty of Medicine, University of Calgary

## Abstract

Pelvic pain is a common condition that is associated with dysmenorrhea and endometriosis.  In some women the severe episodes of cyclic pain change and the resultant pain becomes continuous and this condition becomes known as Chronic Pelvic Pain.  This state can be present even after the appropriate medical or surgical therapy has been instituted.  It can be associated with pain and tenderness in the muscles of the abdomen wall and intra-pelvic muscles leading to severe dyspareunia.  Additional symptoms of irritable bowel and interstitial cystitis are common.   A common sign of the development of this state is the emergence of *cutaneous allodynia* which emerges from the so-called viscero-somatic reflex.  A simple bedside test for the presence of *cutaneous allodynia* is presented that does not require excessive time or special equipment.  This test builds on previous work associated with changes in sensation related to gall bladder function and the viscera-somatic reflex(1;2).

The test is undertaken with the subject s permission after an explanation of how the test will be performed.  *Allodynia* refers to a condition in which a stimulus that is not normally painful is interpreted by the subject as painful.  In this instance the light touch associated with a cotton-tipped applicator would not be expected to be painful.  A positive test is however noted by the woman as suddenly painful or suddenly sharp. The patterns of this sensation are usually in a discrete pattern of a dermatome of the nerves that innervate the pelvis.

The underlying pathology is now interpreted as evidence of neuroplasticity as a consequence of severe and repeating pain with changes in the functions of the dorsal horns of the spinal cord that results in altered function of visceral tissues and resultant somatic symptoms(3).

The importance of recognizing the condition lies in an awareness that this process may present coincidentally with the initiating condition or after it has been treated.  It also permits the clinician to evaluate the situation from the perspective that alternative explanations for the pain may be present that may not require additional surgery.

**Figure:**
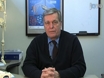


## Protocol

### Introduction

In most cases the areas of *cutaneous allodynia* will be identified in the dermatomes of the abdomen and perineum that innervate the intra-pelvic structures – T11-L1 and S3,4,5 respectively. The term *allodynia* refers to the presence of a painful sensation as a consequence of a stimulus that is not normally painful, in this case a cotton-tipped culture stick.

#### 1. Abdomen

An explanation of the test is undertaken and permission to procede is obtained.
The woman exposes the abdomen from the rib cage to the region of the pubic bone.
A cotton-tipped culture stick is shown to the woman and brushed against her hand to demonstrate how the light pressure will feel.
The woman is asked to note the point at which light pressure applied to the skin becomes sharp as the cotton-tipped culture stick is drawn down the abdominal wall.
The cotton-tipped culture stick is then lightly drawn down the abdomen and the woman will either note the presence of no change in sensation or will note a sudden and discrete sharpness in sensation. The process is repeated until the entire area of *cutaneous allodynia* has been identified. A positive test is defined by the presence of this sudden sharp sensation.
In some cases the areas are large but in others they will be small. They can be unilateral but usually will be bilateral.

#### 2. Perineum

An explanation of the test is undertaken and permission to procede is obtained.
The woman exposes the perineum in the lithotomy position.
A cotton-tipped culture stick is shown to the woman and brushed against her hand to demonstrate how the light pressure will feel.
The woman is asked to note the point at which light pressure applied to the skin becomes sharp as the cotton- tipped culture stick is drawn across the perineum.
The cotton-tipped culture stick is then drawn across the buttocks horizontally in a direction from the lateral aspect of the buttock to toward the anus to cut across the S3 dermatome. The process is repeated on the contralateral side. Additionally the cotton-tipped applicator can be drawn from the region of the labia majora in a directly posterior direction, avoiding the vaginal area. A positive test is defined by the presence of a sudden sharp sensation.
On the perineum, the area usually describes the S# dermatome when positive. Positive results can be seen unilaterally as well but less commonly.

## Discussion

The presence of cutaneous allodynia indicates the woman has had severe pain in the past to the extent that neuropathic changes have occurred.  Although the problem may have originated from dysmenorrhea or endometriosis, a new condition has emerged – Chronic Pelvic Pain.   The original condition may be active or quiescent.   Recognition of this new development is important for patient education and to initiate processes of rehabilitative care.  Recognition of the development of this state may also permit the clinician to explore other options and avoid potentially unnecessary surgery – particularly if the original condition has been treated.
